# Study of Free‐Space Optical Quantum Network: Review and Prospectives

**DOI:** 10.1002/advs.202521923

**Published:** 2026-04-14

**Authors:** Hua‐Ying Liu, Zhenda Xie, Shining Zhu

**Affiliations:** ^1^ National Laboratory of Solid State Microstructures School of Physics School of Electronic Science and Engineering National Key Laboratory of Microwave Photonics and Collaborative Innovation Center of Advanced Microstructures Nanjing University Nanjing China

**Keywords:** free‐space quantum network, ground station, mobile platforms, satellites

## Abstract

The optical quantum network plays an important role in quantum information technology, enabling important applications including quantum communication, distributed quantum computing, quantum remote sensing, etc. Without the fiber connection limitation, free‐space quantum network is free from fiber scattering loss, and enables more flexible quantum network connections. In recent years, rapid progress has been achieved in free‐space quantum networks using fixed ground stations, satellites, and mobile platforms. Fixed ground stations are the primary testbeds for free‐space experiments, and link distances of up to 144 km have been demonstrated between selected locations. Satellites, free from atmospheric turbulence and absorption, further boost the distance to over a thousand kilometers, enabling intercontinental connections. Besides fixed ground stations and satellites, mobile platforms offer highly flexible connections to end users, as fully presented using drones, after preliminary works using balloons, aircraft, helicopters, etc. Here we review the free‐space experiments based on these three platforms, and analyse and compare their characteristics, advantages, and limitations in different scenarios, respectively. In the prospectives, we discuss their unique and complementary roles, and the key challenges and enabling technologies toward the ultimate goal of a practical quantum network, in conjunction with the fiber‐based quantum network.

## Graphical abstract

Free from the constraints of fiber connections, free‐space quantum network enables longer and more flexible quantum network connections. This review summarizes and comparatively analyzes free‐space quantum network experiments based on ground stations, satellites, and mobile platforms. Prospects for space‐air‐ground‐sea integrated quantum network are outlined, highlighting the unique and complementary roles of each platform, together with fiber‐based quantum network.

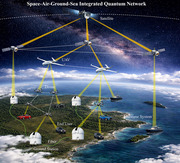

## 1 Introduction

Photons are the natural choice of the carrier of information, either in a classical network or a quantum network, because of their weak interaction with materials and low propagation loss. Fiber‐based networks have achieved great success in classical communication, taking advantage of their low‐loss fiber technology and robustness to the environment. In optical quantum network construction, the fiber network also plays an important role since the very beginning of quantum network demonstration, taking advantage of the well‐developed classical fiber technology. The first fiber‐based quantum network experiments date back to 1993, where Gisin's Group demonstrated a fiber‐based quantum cryptography with a link distance of 1.1 km [1]. In this experiment, they transmitted photon states coded in different polarizations to generate shared secret keys between two remote users, which is called the quantum key distribution. It can guarantee information‐theoretic security, based on some fundamental principles of quantum physics [2]. In the following decades, quantum experiments using different methods and technologies have been demonstrated in the fiber quantum links, with longer distances step by step [3, 4, 5, 6]. In these works, weak coherence states (WCS) were used as the single photon source for QKD, which arise from strongly attenuated laser pulses with average photon number at a single photon level. It results in practical security loopholes, where attacks like photon‐number‐splitting can break the security [7]. In 2003, Hwang et al. proposed the decoy‐state protocol [8], in which the photon‐number‐intensity of the transmitted pulses is varied randomly, so that legitimate users can detect potential eavesdropping and hence solve the security loophole arising from the non‐ideal photon source. After that, different groups [9, 10, 11] have demonstrated decoy‐state quantum key distribution (QKD) in fiber links with distances over 100 km in the same year of 2007. Since then, the development of fiber‐based quantum communication has progressed from experiments to practical applications. Based on the mature fiber optical communication network, numerous backbone fiber quantum communication networks have been realized, including the Beijing‐Shanghai Trunk Line [12], the network linking Moscow and St. Petersburg [13], the DARPA Quantum Network [14], and the EuroQCI Network [15].

Besides the fiber‐based quantum network, the free‐space connection has special significance for the quantum network demonstration. Unlike the classical network, quantum information is highly sensitive to loss, in which the loss from the quantum channel not only reduces the data rate, but also makes it insecure when the loss exceeds the secure threshold. The free‐space optical quantum network can provide low‐loss quantum links that are free of fiber scattering loss, hence can realize longer quantum connections. Besides, the quantum information has to be carried by single photons to end‐users, which calls for a flexible and reconfigurable photon connection, in which free‐space quantum network can be a suitable choice. Hence free‐space quantum network is of special importance and is indispensable besides the fiber‐based network technology. Generally, the free‐space quantum network experiments nowadays are mainly based on three types of platforms, including the fixed ground stations, satellites, and mobile platforms. Table 1 summarizes the key characteristics of these three types of quantum networks, which we will introduce in detail in the following Sections 2, 3, and 4, respectively.

**TABLE 1:** Comparison of QKD in representative free‐space quantum‐network platforms.

Platform	Typical distance	Typical key rate	Cost	Maturity	Functionality	Main challenges
Ground stations	100 m–100 km; up to 144 km [11]	Exceeding 1 Mbit/s [16]	Low‐medium	High	Interface between fiber‐based network and moving platforms; testing of new protocols	Atmospheric turbulence; weather dependence; background noise; line‐of‐sight constraints
Satellites	500–1200 km (direct link) [17]; 4600–12 900 km (trusted relay) [12, 18, 19]	1 kbit/s [17] −10 kbit/s [12]; 1.07 Mbit per pass [18]	High	Low‐medium	Long‐distance links toward global coverage	High cost; payload size, weight, and cost (SWaP‐C) constraints; limited satellite pass time; High tracking accuracy requirement; Space working conditions
Mobile platforms	10 m [20] −100 km [21]	∼100 bit/s −10 kbit/s [20, 22]	Low‐Medium	Medium	Plug‐and‐play links; coverage to end‐users	Payload SWaP‐C constraints; High tracking accuracy requirement in motion; Vibration and fast‐changing working conditions;

*Note*: This table compares the works of WCS‐based QKD for fair comparison.

## 2 Ground‐Based Quantum Network Experiments

For a free‐space quantum network, high‐accurate beam pointing is necessary, because it is the fundamental requirement for the establishment of free‐space optical channels with low loss. Robust optical quantum systems are also needed for prolonged out‐of‐lab tests. It is natural to first demonstrate these technologies on the ground, hence the first free‐space quantum network experiment has been demonstrated using a fixed ground station. Beginning with the preliminary outdoor tests by Jacobs and Franson in 1996 [23], and Buttler et al. in 1998 [24], the distance for point‐to‐point free‐space QKD experiments increased gradually, from hundreds of meters to over 20 km in the following few years [25, 26, 27, 28]. In these works, WCS were distributed to generate quantum keys. Besides such a method, entangled photon states in which a photon pair is non‐classically correlated to each other regardless of the distance separating them [29], can also be used for QKD. In 1991, Ekert proposed an entanglement‐based cryptography [30], which can provide an inherent source‐independent security, drawing attention to the realization of entangled distribution.

**FIGURE 1:**
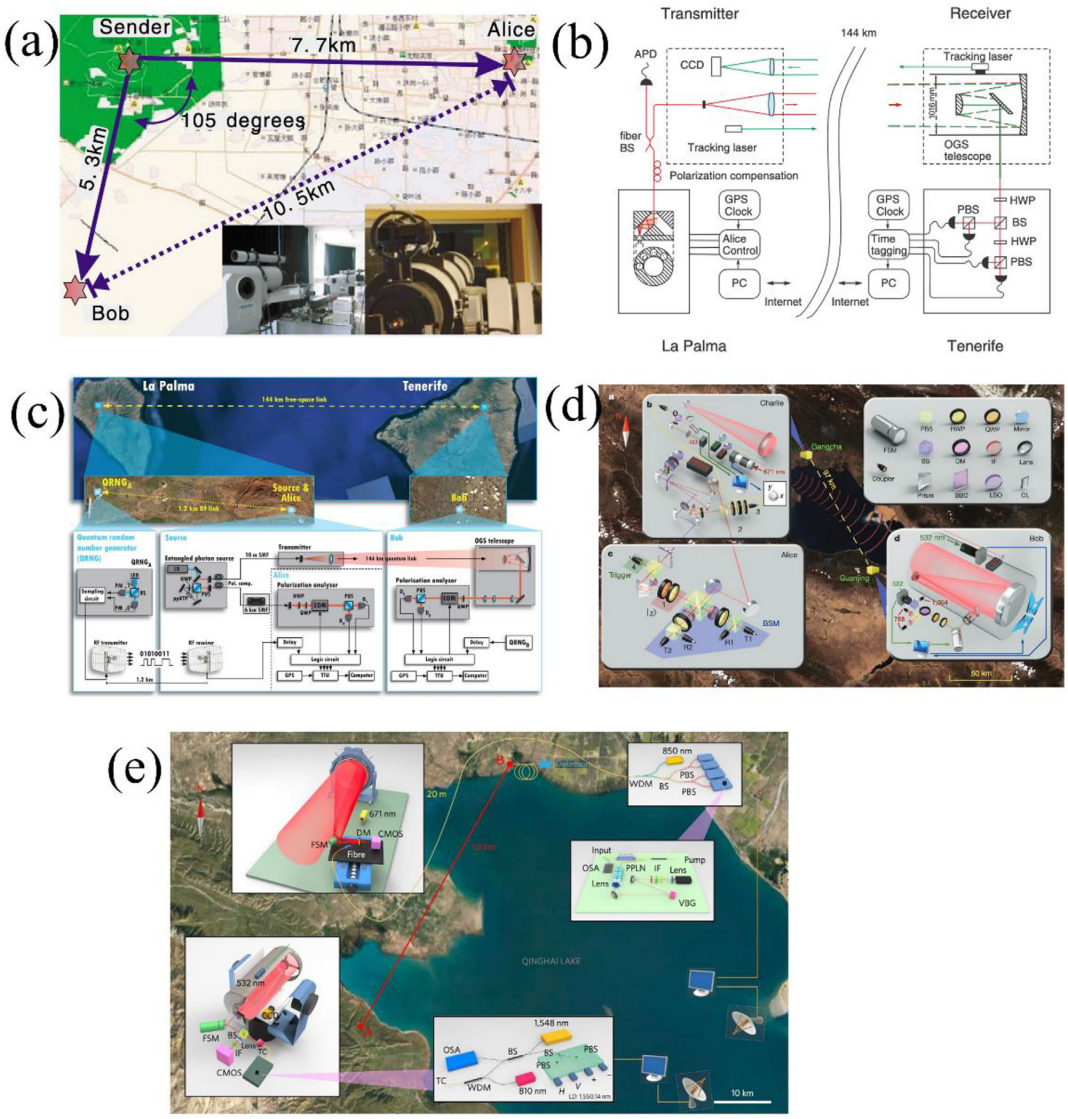
(a) Experimental free‐space distribution of entangled photon pairs over 13 km. Reproduced with permission [40]. Copyright 2005, American Physical Society. (b) Free‐space decoy‐state quantum key distribution over 144 km. Reproduced with permission [11]. Copyright 2007, American Physical Society. (c) Bell's inequality violation experiment. Reproduced with permission [44]. Copyright 2010, National Academy of Sciences. (d) Quantum teleportation over a 97 km free‐space channel. Reproduced with permission [45]. Copyright 2012, Springer Nature. (e) The 53 km QKD experiment in daylight. Reproduced with permission [49]. Copyright 2017, Springer Nature.

Additionally, the distribution of entangled photon states with high fidelity itself is also important. It can be used not only for fundamental quantum mechanical studies, such as tests of Bell's inequality [31], but also for many quantum information applications, such as quantum teleportation [32], distributed quantum computing [33, 34, 35, 36], and quantum metrology [37, 38], etc. In 2003, the first free‐space entanglement distribution was realized by Aspelmeyer et al. over a link distance of 600 m [39]. In 2005, Peng et al. realized a bidirectional entanglement distribution over a total distance of 13 km, which is the first demonstration exceeding the effective atmosphere thickness toward outer space, as shown in Figure 1a [40]. In the same year, Resch et al. realized a 7.8 km free‐space entanglement distribution in Vienna, proving the feasibility for entanglement‐based QKD in inter‐city free‐space links [41].

Taking advantage of the geographical condition with clear atmosphere, Zeilinger's group realized a 144 km free‐space quantum link between the Canary Islands of LaPalma and Tenerife in 2007. Using this link, they realized decoyed‐state quantum key distribution (Figure 1b) [11], and entanglement distribution in the same year [42]. In 2009, the same group successfully demonstrated an entanglement distribution experiment in high‐loss conditions using the same free‐space link, with link loss up to 64 dB [43]. In 2010, they further presented a Bell's inequality violation experiment, closing the locality loophole and the freedom‐of‐choice loophole at the same time, as shown in Figure 1c [44]. In 2012, Yin et al. also realized entanglement distribution in a bidirectional link across Qinghai Lake with a distance of 101.8 km [45]. In 2013, Cao et al. applied entanglement distribution in quantum communication, and demonstrated a free‐space entanglement‐based quantum key distribution experiment between two sites that are 15.3 km apart [46].

With the capability of high‐fidelity entanglement state distribution, a lot of experiments for quantum teleportation have also been demonstrated in free space. In 2004, Ursin et al. realized quantum teleportation in a 600 m free‐space link across the Danube River [47]. In 2010, Jin et al. realized quantum teleportation over the Great Wall of China at a free‐space link distance of 16 km, which is based on a full Bell‐state measurement (BSM), with real‐time information transmission ensured by an active feed‐forward technique. In 2012, the link distance was further extended over a 97‐kilometre one‐link free‐space channel across Qinghai Lake by the same group, with quantum teleportation achieved using a multi‐photon entangled source, as shown in Figure 1d [45]. In the same year, quantum teleportation was also demonstrated by Ma et al. [48], in the 143 km free‐space channel between the two Canary Islands of La Palma and Tenerife. With these achievements, photon states can be transferred over long free‐space quantum links, for application in large‐scale quantum communication and distributed quantum networks.

At the early stage, the development of ground‐based free‐space quantum network experiments focused on the demonstration of simple optical quantum experiments, such as quantum key distribution and entanglement distribution at night, with advances mainly in longer distances. Thereafter, more fancy experiments have been conducted, which may be more suitable for the practical application of the free‐space quantum network in the future.

The daytime operation capability is crucial for full‐time coverage of the free‐space quantum network, yet the strong sunlight background noise brings a great challenge for its realization. In the early works, efforts have been made in spectral, temporal, and spatial filtering, for the demonstration of quantum key distribution at daytime with distances from hundreds of meters to over 1 km [26, 50, 51]. In 2017, Liao et al. realized free‐space QKD over a distance of more than 53 km at daytime, as shown in Figure 1e [49]. In this work, photons were operated at the telecom band to reduce the strong background noise from solar radiation, and low‐dark‐count up‐conversion single photon detectors (SPD) were developed for their detection. As a result, QKD was successfully realized in free space during the local time from 15:30 to 17:00 on sunny days. To improve the link coupling efficiency, they adapted the adaptive optics (AO) in their system in the following year, which is a technique that compensates the atmospheric turbulence‐induced wavefront distortions dynamically using wavefront modulators such as deformable mirrors or spatial light modulators. Based on such a strategy, they successfully improved the signal‐to‐noise ratio (SNR) and realized free‐space QKD in an 8 km urban daylight link [52]. In 2021, Gruneisen et al. also used AO to improve the coupling efficiency of the free‐space link and realized quantum key distribution during daylight with a distance of 1.6 km [53]. In 2018, Ko et al. realized QKD under daylight in a 275 m free‐space quantum link and quantized the influence of filtering techniques in the spectral, temporal, and spatial domains on the quantum bit error rate (QBER) and secure key rate [54]. In 2021, Avesani et al. used integrated silicon chips as the encoder and realized daylight QKD in a 145 m free‐space urban link [55]. In 2023, the quantum dot photon source has also been used for daytime QKD by Basset et al., with a link distance of 270 m in free space [56]. In 2024, Cai et al. developed a 625 Mhz repetition rate QKD system with spectral filtering near the Fourier transform limit, and realized all‐day operation over a 20 km free‐space link, paving the way for all‐day applications [57].

In these experiments, polarization encoding is chosen because of its resistance to the atmospheric turbulence in free‐space channels, in which quantum information is coded on the polarization of the photons. Besides that, other degrees of freedom (DoFs) have also been used in free‐space experiments in recent years.

**FIGURE 2:**
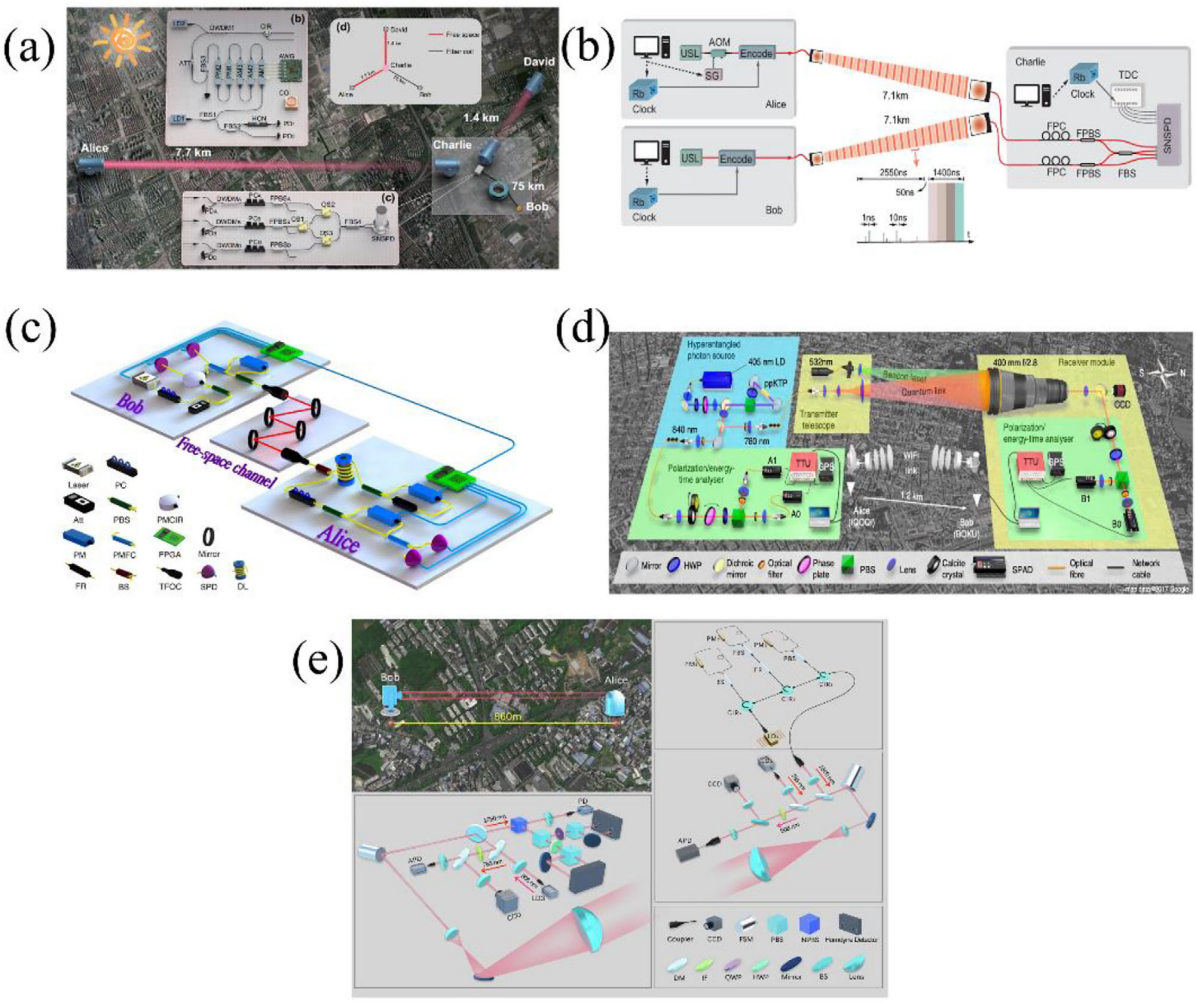
Setup of free‐space and fiber‐integrated MDI‐QKD. Reproduced with permission [63]. Copyright 2023, American Physical Society. (b) Set up of the free‐space TF‐QKD [64]. Copyright 2025, arXiv preprint. (c) Schematic diagram of free‐space QSDC system. Reproduced with permission [66]. Copyright 2020, Optica Publishing Group.(d) Illustration of the high‐dimensional entanglement distribution experiment. Reproduced with permission [77]. Copyright 2017, Springer Nature. (e) Setup of free‐space CV‐QKD. Reproduced with permission [89]. Copyright 2025, Springer Nature.

For example, time‐bin encoding is an emerging choice for quantum information technologies nowadays, which codes quantum information in the discrete temporal modes of photons. The most common choice for time‐bin coding nowadays is phase encoding, which uses the relative phases between photons in early and late temporal modes as carriers for quantum information. In 2025, time‐bin encoded BB84 QKD has been realized in free space by many groups from Canada, Italy, and China independently [58, 59, 60]. Based on time‐bin encoding, many new QKD protocols have also been applied in free‐space. In 2020, Cao et al. realized the first free‐space measurement‐device‐independent QKD (MDI‐QKD) over a 19.2‐km urban atmospheric channel [61]. Such protocol can remove security vulnerabilities associated with detection devices by performing Bell‐state measurements at an untrusted intermediate node, thus offering enhanced security [62]. In 2023, Li et al. further realized MDI‐QKD in a hybrid link integrating free‐space and fiber channels together, as shown in Figure 2a [63]. Recently, another interesting protocol, the twin‐field QKD (TF‐QKD), has also been realized in free space by the same group. It was achieved by two free‐space channels over the urban atmosphere, with a total distance of 14.2 km, as shown in Figure 2b [64]. Compared to MDI‐QKD, which relies on two‐photon interference, the realization of TF‐QKD only requires single‐photon interference. So that it can elevate the dependence of key rate on loss from linear to square‐root while maintaining the MDI security, hence extending the maximum achievable link distance [65].

In 2020, Long's group realized another interesting quantum communication method, the quantum secure direct communication (QSDC) in free‐space quantum network using phase encoding, as shown in Figure 2c [66]. QSDC uses quantum states as carriers for secure communication directly without the requirement of generating secret keys first, which has been proven to be information‐theoretically secure [67, 68, 69, 70, 71, 72]. In this work, they achieve an information transmission rate of 500 bps over a 10 m free space channel with a mean quantum bit error rate of 0.49% ± 0.27%, proving the feasibility of QSDC in free space. In 2022, the same group further realized QSDC in a fiber and free‐space hybrid quantum network [73]. A three‐node secure‐repeater network is constructed, comprising a 10 km fiber link and a 2‐meter free‐space link, providing an important architecture for the quantum network.

Besides the binary phase encoding, the high dimensionality of the temporal modes can also be harnessed for free‐space quantum network, which can greatly enlarge the quantum information capacity of photons. Such high‐dimensional QKD has already been demonstrated in the fiber‐based QKD works, which can be applied in free‐space quantum network to improve the key rate in the future [74, 75, 76]. Besides coding in a single DoF, hyperentanglement photon states are also a good choice to improve the quantum information capacity of photons, in which the photon pair is entangled in multiple degrees of freedom. In 2017, hyperentanglement distribution encoded in both polarization and energy‐time bases was also demonstrated in a 1.2‐km‐long free‐space link across Vienna by Steinlechner et al., as shown in Figure 2d [77]. With the application of high‐dimensional entanglement, enhanced information capacity and noise resilience can be achieved in free‐space quantum links. In 2023, the same group distributed high‐dimensional energy‐time entangled photons over a 10.2 km free‐space quantum network, and used the distributed photons to realize QKD [78]. Additionally, orbital angular momentum (OAM) has also been implemented in free space quantum network [79, 80]. It codes quantum information in the helical wavefront of photons, which is also a promising option for high‐dimensional encoding [81]. Progress has also been made in the theoretical research on OAM‐encoded QKD to improve the photon collection efficiency of the system [82].

These aforementioned works are based on the discrete variable encoding. Besides that, the quantum information can also be encoded and transmitted in the continuous variable (CV), which is called the CV‐QKD. In this protocol, the quantum signals are typically implemented in quadrature variables of coherent state of light, and measured using homodyne or heterodyne detection rather than single‐photon detection. Such method can provide high‐key‐rate, and low‐cost communication with strong resistance to background light noise [83]. Recently, several studies on key techniques and experimental demonstrations have also been conducted on free‐space CV‐QKD [84, 85, 86, 87, 88]. In 2025, Zheng et al. realized CV‐QKD in free space during the day and even on rainy days, with a secure key rate (SKR) of more than 100 kbps [89], showing the potential of CV‐QKD for practical application in the future, as shown in Figure 2e. Yin et al. further explore the possibility of realizing CV‐QKD on unmanned vehicles (UVs) like drones. As a proof‐of‐principle demonstration, they achieved passive‐state‐preparation CV‐QKD during daytime and night in a high‐attenuation environment between two buildings, providing a promising solution for the UV secure communication system with cost‐effectiveness, integration, and adaptability [90]. Recently, they further extended the link distance to 9.6 km in a maritime atmospheric channel, marking an important step for the practical application of CV‐QKD in free‐space quantum network [91].

In 2021, Hu et al. also achieved polarization‐encoded BB84‐based QKD over an air‐water channel. To achieve higher transmittance, the photons were operated at 450 nm. Secure keys were generated successfully against high loss with a low quantum bit error rate of less than 2.5% for underwater links with a distance up to 30 m, demonstrating its capability for real‐life air‐sea quantum‐communication tasks [92]. With such technology, quantum links can be established to underwater vehicles, which is useful, besides the free‐space quantum links in the atmosphere, for free‐space quantum network.

To summarize and provide a more systematic understanding of protocol selection in different free‐space environments, Table 2 presents a comparative overview for representative QKD protocols in free‐space quantum network, including BB84, MDI‐QKD, TF‐QKD, and CV‐QKD. Generally, even though long‐distance quantum network between fixed ground stations is weather‐dependent, and the longest quantum network is realized at very special experimental locations for stable transmission. These works prove that free‐space channel can be used to achieve lower link loss compared to the fiber, for realizing longer link distances, which also establishes the foundation for the quantum network in space.

**TABLE 2:** Comparison of major QKD protocols in free‐space quantum communication. η denotes the transmittance of the channel.

Protocol	Characteristics	Key rate scaling	Maturity	Complexity	Representative demonstrations	Advantages	Limitations and challenges
BB84	Prepare‐measure	Rate∼η	Highest	Low	Demonstration in ground station, satellites, and mobile platforms	Simple implementation; mature technology	Detector side‐vulnerabilities
MDI‐QKD	Measurement‐independent security; two‐photon interference	Rate∼η^2^	Medium	High	19.2‐km free‐space urban channel [61]	Detector side‐attack immunity	Complex synchronization; Low‐key rate
TF‐QKD	Measurement‐independent security; single‐photon interference	Rate∼η^1/2^	Low	Highest	14.2‐km free‐space urban channel [64]	Highest loss tolerance; suitable for extending distance	Phase stabilization challenge in free‐space channels
CV‐QKD	Continuous variable‐encoding; homodyne/heterodyne detection	Rate∼η	Medium	Medium ‐High	9.6‐km maritime atmospheric channel [91]	High key rate; compatible with classical coherent communication technology	Sensitive to excess noise; limited long‐distance robustness

## 3 Satellite‐Based Quantum Network Experiments

Compared to the free‐space quantum network within the atmosphere, the space channel benefits from the near‐vacuum environment, which is free from scattering loss and atmospheric turbulence. Hence for a large‐scale quantum network (typically hundreds to thousands of kilometers), the satellite‐based free‐space quantum network might be a suitable choice, just like the satellite‐based network in classical information technology.

**FIGURE 3:**
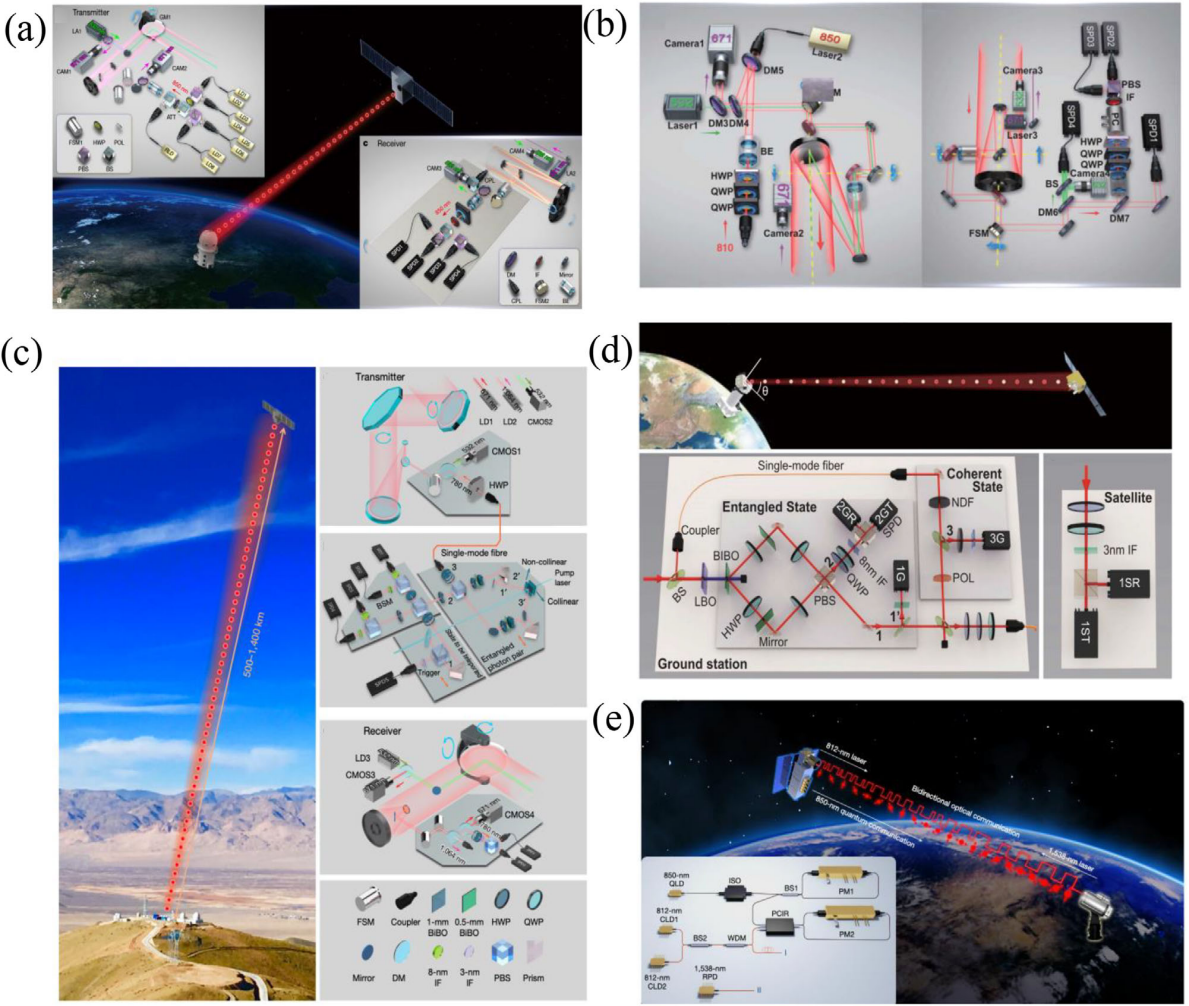
(a) Satellite‐to‐ground QKD over 500‐1200 km downlink. Reproduced with permission [17]. Copyright 2017, Springer Nature. (b) The transmitters, receivers, and acquisition, pointing, and tracking (APT) system of satellite‐based entanglement distribution. Reproduced with permission [97]. Copyright 2017, American Association for the Advancement of Science. (c) Ground‐to‐satellite quantum teleportation. Reproduced with permission [99]. Copyright 2017, Springer Nature. (d) Schematics of experimental test of event formalism in Earth's gravitational field. Reproduced with permission [100]. Copyright 2019, American Association for the Advancement of Science. (e) Real‐time satellite‐to‐ground QKD with Jinan‐1. Reproduced with permission [18]. Copyright 2025, Springer Nature.

Aiming at this purpose, efforts have been made by many groups around the world for satellite‐based optical quantum experiments [93, 94, 95, 96]. In 2016, Pan's team launched the world's first quantum satellite, the Micius. Using this satellite, they realized the first satellite‐to‐ground QKD over 500–1200 km downlink, implementing the polarization‐encoded BB84 protocol, as shown in Figure 3a. In this work, they successfully generated secure keys at kilobit‐per‐second rates under channel losses exceeding 40 dB, confirming the practicality of QKD in long‐distance, high‐loss, and high‐dynamic space‐to‐ground channels [17]. In 2018, Liao et al. used the Micius satellite as a trusted relay to established secret key between itself and different ground stations. In this way, secret keys were created between China and Europe at locations separated by 7600 km on Earth, which were then used for intercontinental quantum‐secured communication, including one‐time‐pad image transmission and an intercontinental video conference [19]. In 2021, an integrated space‐to‐ground quantum communication network combining the Micius and a large‐scale fiber network of more than 700 fiber QKD links was demonstrated. This network can achieve an average secret‐key rate of 47.8 kbit/s for a typical satellite pass, covering users in the network with a total distance up to 4600 km [12].

Based on such a satellite‐to‐ground link, entanglement distribution has also been demonstrated. In 2017, Yin et al. realized the first satellite‐based entanglement distribution with the polarization‐entangled photon pair generated on board and separately distributed to two ground stations over 1000 km apart, as shown in Figure 3b. In this work, they performed the Bell inequality test between the distributed photon pair, which is a quantitative test to distinguish quantum correlations from classical local hidden‐variable theories [31]. The observation of a violation of the Bell inequality by 2.37 ± 0.09 proves that entanglement was preserved, despite atmospheric turbulence and long‐distance channel losses in the satellite‐to‐ground link. This milestone marked the first demonstration of entanglement distribution on an intercontinental scale [97]. Based on the same configuration, they further realized the first entanglement‐based QKD between two ground stations (Delingha and Nanshan) 1120 km apart, with a final secure key rate of approximately 0.12 bit/s [98].

Upon these experiments, entanglement photon states can be transferred from the satellite to remote terminals with high fidelity. In 2017, Ren et al. successfully demonstrated ground‐to‐satellite quantum teleportation over distances ranging from 500 to 1400 km using the Micius satellite, establishing a foundation for a satellite‐based quantum internet, as shown in Figure 3c [99]. The entanglement distribution has also been used for fundamental quantum mechanical experiments. In 2019, Xu et al. demonstrated a quantum optical test of a gravitationally induced quantum decoherence model using the Micius satellite. By satellite‐to‐ground distribution of time‐energy entangled photons, no evidence for decorrelated effects was observed, which is consistent with the standard quantum theory, as shown in Figure 3d [100]. This experiment may help shed light on the interplay between quantum theory and gravity.

With advances in integration and miniaturization, a more compact microsatellite, Jinan‐1, was launched in 2022. By improvements in the integrated design of optical components, drive electronics, and the fusion of optical‐fiber components, etc., the weight of the quantum payload was only 22.7 kg. Through an optical‐mechanical integrated design, the receiver at ground had also been minimized from a bulky astronomical telescope to a portable terminal, with weight reduced from approximately 13 000 kg to a mere 100 kg. In 2025, Pan's group used Jinan‐1 as a trusted relay to demonstrate real‐time satellite‐to‐ground QKD, as shown in Figure 3e. It can generate 1.07 Mbit of secure keys in a single satellite pass, which was used to support one‐time‐pad‐based image transmission between China and South Africa at locations separated by over 12 900 km on Earth. Such micro quantum satellite demonstrates the potential for establishing lightweight quantum satellite constellations in the future [18].

Based on these works, it has been proven that satellites can establish low‐loss quantum links over 1000 km. So far, all the experiments are based on satellite‐to‐ground links, and satellite‐to‐satellite links are now in progress. Once established, it can take full advantage of the satellite‐based quantum network, providing network service similar to the classical satellite‐based network, for quantum connection to multiple users all around the world.

## 4 Mobile‐Platform‐Based Quantum Network Experiments

In a classical information network, connection to end‐users is achieved by cellular wireless communication technology rather than optical communication technology, which can offer broadcasting‐type mobile communication within its coverage range. However, for quantum network connection, it requires sending single photon states directly to end‐users, which is fundamentally different from the classical network. To satisfy such a requirement, the reconfigurability of free‐space optical quantum network is very important, which requires the establishment of a mobile platform quantum network using moving platforms as hosts.

**FIGURE 4:**
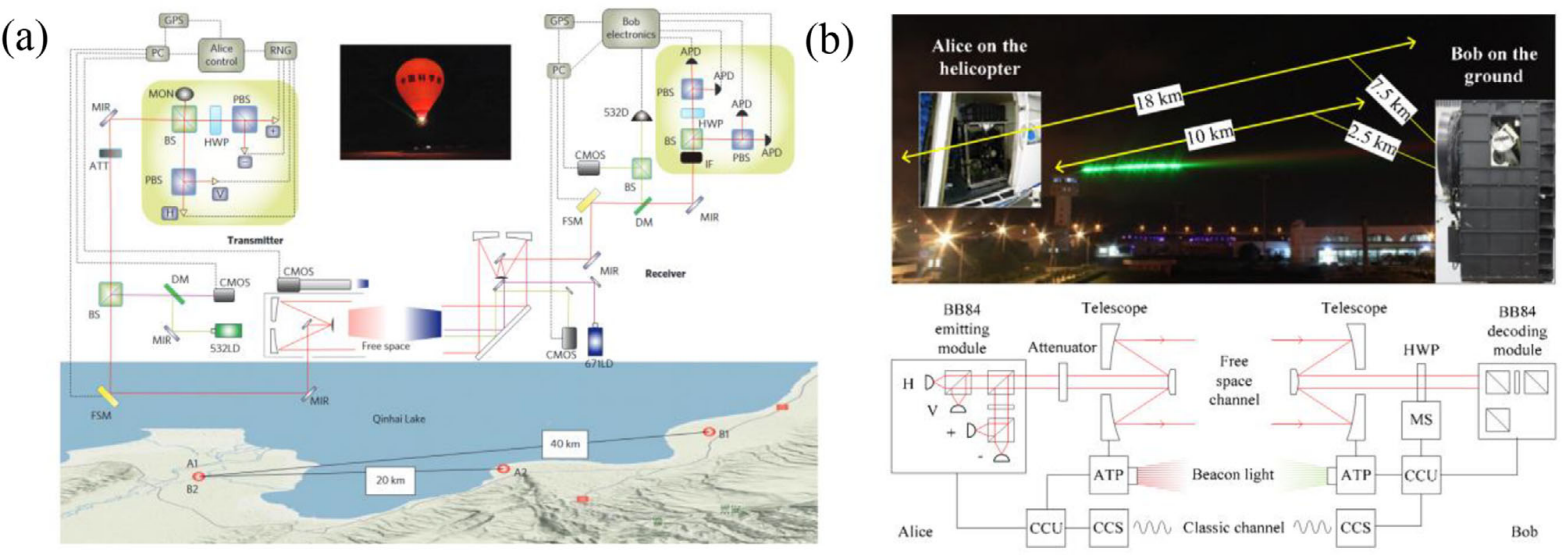
(a) Structure diagram of hot air balloon quantum key distribution. Reproduced with permission [21]. Copyright 2013, Springer Nature. (b) Helicopter air‐ground quantum key distribution experiment. Reproduced with permission [102]. Copyright 2014, Optica Publishing Group.

In the early experiments, researchers have used different moving platforms to establish the free‐space quantum links. In 2013, Wang et al. demonstrated three independent free‐space experiments with a decoy‐state QKD system operating on a moving turntable, a floating hot‐air balloon, and with a high‐loss channel over a link distance of 40, 20, and 96 km, respectively, as shown in Figure 4a [21]. These experiments demonstrate that their QKD system could generate secure keys with a low quantum bit error rate (QBER) even under extreme conditions, with angular velocities up to 21 mrad/s and channel losses as high as 50 dB, proving its potential for satellite‐to‐ground QKD. In the same year, Nauerth et al. reported the first successful implementation of QKD from a flying aircraft to a ground station [101]. In this work, they overcame critical challenges associated with rapidly moving platforms, such as real‐time tracking and polarization compensation, and achieved a secure key rate of 145 bit/s. To solve the potential polarization‐basis‐deviation problem in satellite, Zhang et al. from the Chinese Academy of Sciences employed a helicopter‐mounted transmitter to simulate satellite motion. In this work, they experimentally demonstrated the reliability of the polarization compensation system under rapid motion, attitude fluctuations, and atmospheric disturbances, as shown in Figure 4b [102]. In 2015, Bourgoin et al. reported QKD from a stationary transmitter to a moving receiver ∼650 m apart, and achieved a secure key rate of approximately 40 bit/s [103]. The receiver is located on a tracking system traveling at an angular speed equivalent to that of a 600 km altitude satellite, thereby supporting the feasibility test for satellite‐based QKD.

The works above have demonstrated the capability for air‐to‐ground free‐space quantum links with moving platforms, with the primary aim of a feasibility test for satellite‐based quantum links. These platforms were operated either in a relatively fixed location or an artificial orbit simulating the characteristics of the satellite, using relatively large platforms, which might not be suitable for mobile applications. For a practical mobile quantum network that can provide a plug‐and‐play connection to end‐users, free‐moving hosts with a small size are needed. Recently, the boosting development of the low‐altitude mobile platforms, typically the drones, provides an ideal platform for such a mobile quantum network. By leveraging the flexibility, mobility, and cost‐effectiveness of drones, fast‐deployed and reconfigurable quantum links can be established on demand. However, such mobile features limit capacity and higher maneuverability, which brings in challenges quite different from these satellite‐oriented experiments. It calls for breakthroughs not only in the quantum payloads, such as the quantum source, the QKD modules, etc., but also in the acquisition, pointing, and tracking (APT) systems, which are used to establish and maintain precise optical alignment between two distant terminals.

**FIGURE 5:**
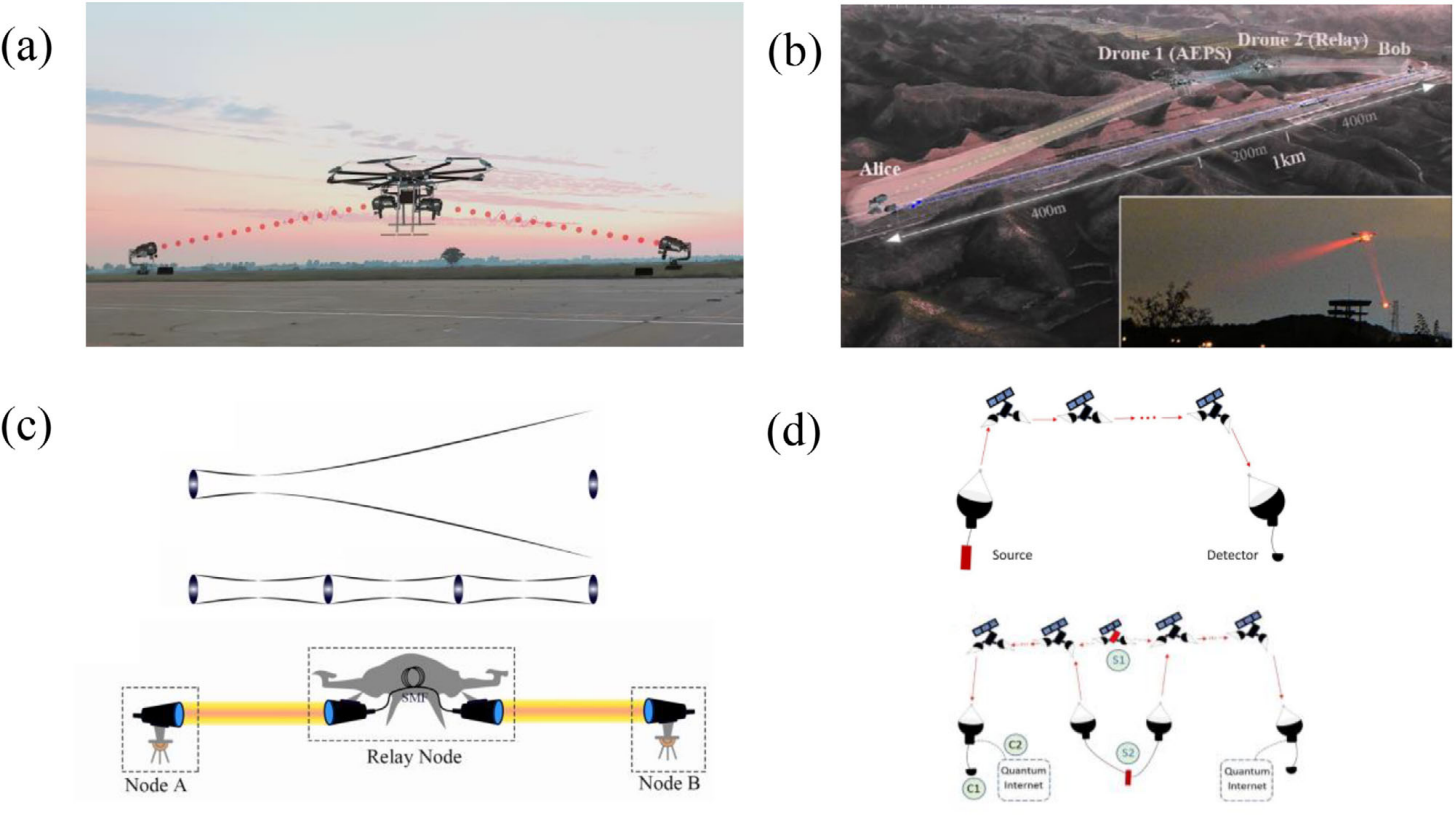
(a) Schematic of the APT system in drone‐based entanglement distribution experiment. Reproduced with permission [104]. Copyright 2020, Oxford University Press. (b) Quantum network and physical realization with mobile drone‐based nodes. Reproduced with permission [105]. Copyright 2021, American Physical Society. (c) Illustration of the experimental setup for the relayed entanglement distribution. Reproduced with permission [105]. Copyright 2021, American Physical Society. (d) Schematic of the all‐satellite quantum network. Reproduced with permission [106]. Copyright 2023, American Physical Society.

In 2020, Liu et al. demonstrated the first drone‐based entanglement distribution experiment [104]. In this work, they developed an airborne polarization entangled photon source, which weighs only 468 g while maintaining high brightness of 2.4 × 10^6^ bps, visibilities over 97.0%, and a near‐theoretical limit CHSH S parameter of 2.725 ± 0.017. A two‐stage airborne APT system was also developed, which achieves a tracking accuracy of ∼6 µrad under flight conditions while controlling the weight to only 3.7 kg. Loading them on a home‐made octocopter, a 200 m drone‐to‐ground quantum link is established. And Bell inequality violation was observed between the two distributed ground stations under various weather conditions, including clear night, daytime, and rainy night. These results demonstrated the reliability of such drone‐based quantum link for multi‐weather quantum operation, as shown in Figure 5a [104].

In 2021, they further established the first air‐to‐air quantum link between two flying drones, and realized optical‐relayed entanglement distribution in free space, with the Clauser‐Horne‐Shimony‐Holt (CHSH) S‐parameter measured to be 2.59 ± 0.11. As shown in Figure 5b, entangled photons are generated and transmitted from one drone, and then coupled and recollimated by a second drone for retransmission to the ground station. In free‐space quantum network, the diffraction loss, also known as the geometric loss, is an important component for propagation loss, which arises from the optical beam diffraction. It sets a limit for link distance, in which, for longer links, larger optical aperture antennas are needed. With the optical relayed configuration in this work, such a fundamental problem can be solved, as the profile of the single photon can be reshaped at each relay node, hence reducing the diffraction loss, as shown in Figure 5c. Based on this technology, the team successfully increased the total link distance to 1 km, without increasing the optical aperture and weight of the APT system. In these experiments, they conducted experiments across multiple locations in China, including Nanjing, Lanzhou, and Shijiazhuang, etc. Thus, it demonstrated its robustness for application in diverse weather conditions, such as varying temperatures, humidity levels, and altitudes. Upon these works, the building blocks for multi‐node mobile quantum links toward an even mobile quantum network with complex structures have been developed [105].

Interestingly, after being proposed first for the mobile platform, a similar idea was proposed by the Goswami et al. [106], for satellite‐to‐satellite and satellite‐to‐ground quantum links in 2023. As shown in Figure 5d, the core of this architecture is to regard the telescopes on satellites as “satellite lenses”. By carefully designing the spacing and focal length of these “satellite lenses”, the light beam can be periodically focused, thereby achieving low‐diffraction‐loss satellite‐to‐satellite and satellite‐to‐ground information transmission.

**FIGURE 6:**
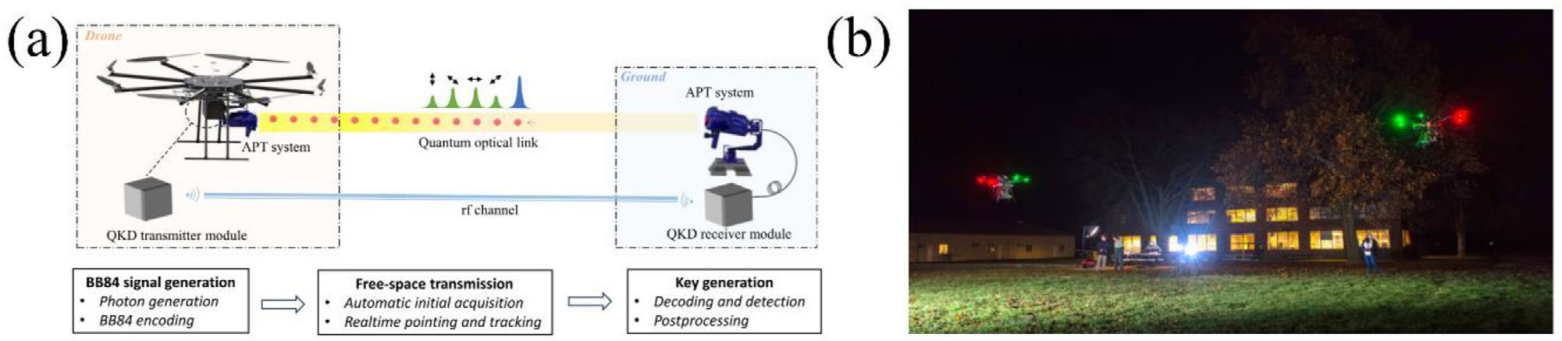
Schematic of drone‐based QKD performed by (a) Nanjing University [22] and (b) University of Illinois Urbana‐Champaign [20]. Copyright 2025, under the terms of the Creative Commons Attribution 4.0 International License.

Later, in 2024, the same team realized the first drone‐based quantum key distribution, using polarization‐encoded BB84 protocol, as shown in Figure 6a [22]. A 1.5 kg airborne QKD transmitter module was developed, co‐packaging the micro‐optics‐based and printed‐circuit‐board‐integrated control electronics in a box. A 5 kg APT system was also developed, which was capable for automatic acquisition to establish a quantum optical link within 30 s. With the stable in‐motion polarization state transmission technology, real‐time secret keys were generated during both night and day over a 200 m ground‐to‐air free‐space link, with an average secure key generation rate over 8 kHz. Based on the capability of secret key distribution using a drone, wireless communication can be expected with enhanced security in the quantum approach between mobile nodes toward a network. In 2025, Conrad et al. also accomplished QKD in mobile links, with a link distance of ∼10 m and an average secret key rate of 8.5 kbps for the hovering drone‐to‐drone link, as shown in Figure 6b. Other types of mobile links have also been demonstrated with the mobile platforms travelling parallel to each other, in configurations including drone‐to‐vehicle, slow‐moving vehicle‐to‐vehicle and fast‐moving vehicle‐to‐vehicle with speeds of 16, 8, and 113 km/hr, and secret key rate of 1.6, 20.0, and 2.5 kbps, respectively [20].

**FIGURE 7:**
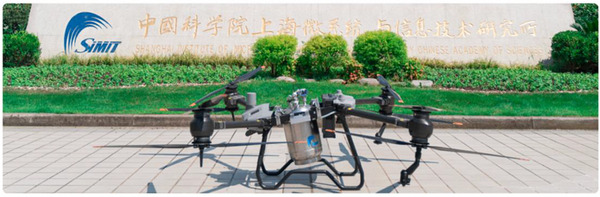
Photo of drone‐based superconducting nanowire single‐photon detection system [107]. Reproduced with permission [107]. Copyright 2025, Optica Publishing Group.

Photon detection with high efficiency and low noise is also essential for free‐space quantum links. For previous experiments in satellites and mobile platforms, the silicon SPDs operating in the visible band were the primary choice because of their compact size and light weight compared to other SPDs. In 2025, Ma et al. from Shanghai Institute of Microsystem and Information Technology (SIMIT) successfully deployed another type of SPD, the superconducting nanowire single‐photon detectors (SNSPDs) on a flying drone, as shown in Figure 7 [107]. In this work, they developed a compact and robust SNSPD system, using a miniature liquid helium dewar and a high‐performance NbTiN superconducting nanowire chip. The total weight for the system is successfully reduced to approximately 12 kg, while maintaining a detection efficiency of 91.8%. It resolves the application limit arising from the traditional bulky and power consumption refrigeration system [108, 109], providing a high detection efficiency, low dark count, broad response wavelength range, and high saturation count rate choice for SPD in mobile quantum network.

Besides these works, more and more attention has been attracted to mobile quantum network, and different types of technologies toward drone‐based applications have been studied. In 2023, Tu et al. proposed a small‐sized and lightweight airborne APT system weighing less than 8 kg, and tested its performance in a 7 km air‐to‐ground link, which could be used to support drone‐based QKD in the future [110]. In the same year, Deng et al. also studied the boundary effects for QKD in air‐to‐air links, which can be used to evaluate the revolution of quantum states for high‐speed flying vehicles in the future. In 2025, Huang et al. studied clock synchronization using Global Navigation Satellite System (GNSS) timing and entanglement‐based timing correction. They achieved 24 ps RMS synchronization without requiring a precision reference clock, which might be used for drone‐based time synchronization in the future [111].

These works prove the feasibility and provide the building block for the mobile quantum network. With more strategy innovation and technology breakthrough, a mobile quantum network that does not rely on pre‐installed fiber or fixed‐orbit satellite can be established, for fast‐deployed connection to end‐users on demand without time or location limitation.

## 5 Outlook

### 5.1 Platform‐Specific Outlook

Generally, low loss and flexibility are the key advantages of the free‐space quantum network. Different from the fiber‐based optical quantum network, the free‐space quantum network can be demonstrated with three major types, using ground stations, satellites, and mobile platforms. The ground‐based quantum link is relatively easy to realize, hence can be the first choice for experiments of new protocols, technologies, etc. for quantum key distribution, entanglement distribution, and quantum teleportation. Besides that, more applications can also be explored in such a quantum link, such as distributed quantum computing [33, 34, 35, 36], remote quantum sensing [112, 113], quantum identity authentication [114, 115], quantum time synchronization [116, 117], etc.

For the satellite‐based quantum link, besides the satellite‐to‐ground quantum links, satellite‐to‐satellite links are also needed, to fill the building block for a satellite constellation for broader coverage. Medium‐Earth‐orbit‐to‐geosynchronous‐orbit satellites will also be used, providing even longer service and broader coverage toward global scale. This type of quantum link is not only planned to be applied for quantum communication, but also for fundamental tests of quantum physics on a large scale, and for more applications such as the high‐precision time‐frequency transfer [118] and the establishment of an optical frequency standard using satellite‐based optical clocks.

For mobile‐platform‐based quantum links, more efforts will be taken to release their full potential in flexibility and reconfigurability. By the development of technologies, including communication in moving, link automatic establishing and fast routing technologies, etc., a plug‐and‐play mobile quantum link can be expected. Benefiting from the various drones, such a link can be established on different scales. In the low‐altitude area, small multirotor drones can be used for establishing 10 km‐level links for metro coverage. While in the high‐altitude area, fixed‐wing drones can be used for establishing longer links with a distance over 100 km for national or transnational connections. The hosts can also be other mobile platforms, such as cars, boats, airships, etc. for a broader coverage satisfying the requirements of end‐users in different scenarios.

In the future, advanced technologies, that have been demonstrated or are ongoing at the ground stations, can also be extended to satellite‐ and mobile‐platform‐based quantum network experiments. In particular, time‐bin encoding becomes a promising alternative for high‐maneuverability platforms. So far, moving‐platform experiments are predominantly based on polarization encoding, because of its robustness in long‐distance free‐space transmission and relatively simple implementation. On the other hand, the time‐bin encoding, which has been widely used in fiber quantum networks, was merely explored in free space, mainly because turbulence might degrade the fidelity of the time‐bin states and phase‐sensitive interferometric devices are challenging to implement on moving platforms. However, because relative motion between terminals results in misalignment of polarization coordinate frame, high‐fidelity polarization compensation is necessary for the polarization encoding in free space, which becomes increasingly difficult for highly maneuverable platforms. For this problem, the time‐bin encoding might be a fundamental solution, as it is intrinsically insensitive to polarization variations. The latest ground‐based experiments [58, 59, 60] have demonstrated that time‐bin quantum states can maintain coherence after transferring in free space. With the development of robust time‐bin devices or even time‐bin circuits, such DoF can be expected to be applied in moving platforms, especially high‐maneuverability platforms in the future. Adoption of time‐bin encoding may also facilitate the implementation of more loss‐tolerant protocols like MDI‐QKD, TF‐QKD, to enhance the network robustness for link loss and harsh weather. Besides the time‐bin encoding, other high‐dimensional DoFs like OAM, frequency bin can also be used, to improve the photon capacity and key rate in the moving platforms.

Besides individual principle innovations and technology breakthroughs, these three types of free‐space quantum links also need to be combined organically toward a network in the future. The ground stations can not only be the proper receiver for the satellites and mobile platforms, but also an ideal relay for connection between the free‐space quantum network and the fiber‐based quantum network. The satellite constellation is a suitable choice for the establishment of a long‐distance network in space. And the mobile platforms blur boundaries among them. The ground station can also be very portable or even loaded on moving vehicles, for a fast‐deployed free‐space quantum network to ground end‐users. It can also fill the blank between the satellite and the ground‐based quantum links, for full‐time all‐location coverage toward a practical quantum network. To make such kind of free‐space quantum network become true, several key enabling technologies are important, which we will summarize in the following section.

### 5.2 Key Enabling Technologies

#### 5.2.1 Quantum Payloads

First, the compact and light‐weight quantum payloads are the common requirement for satellite‐based and mobile quantum network, which require breakthroughs in integration and miniaturization. So far, the quantum payloads, despite the size, weight, and performance, are developed using bulk optics. With the fast development of photonic chips in recent years, it can be expected that the quantum payloads will be integrated on a chip, which can bring revolution in its size, weight, power consumption, and reliability for practical applications. At present, the quantum chips based on silicon‐based platforms are one of the most common choices, mainly because such chips can be fabricated using CMOS‐compatible technology. In such a platform, various quantum chips have been developed, from single‐function devices like a photon source, to multifunction chips such as the QKD encoding/decoding chip. Such a platform provides a promising candidate for fully functional quantum communication chips, for accomplishing photon generation, manipulation, and detection.

**FIGURE 8:**
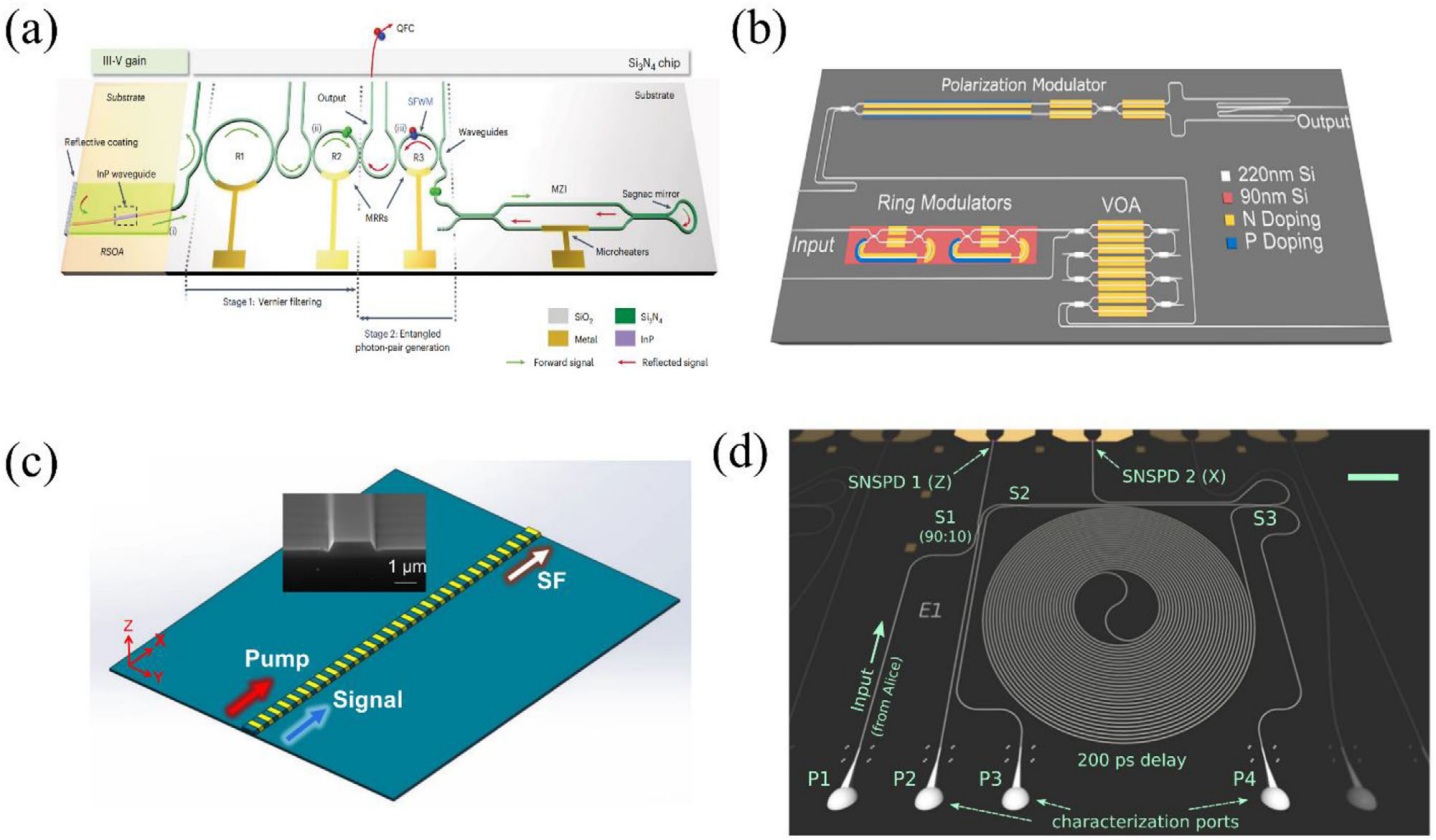
(a) A laser‐integrated photonic quantum source of frequency‐bin entangled photon pairs. Reproduced with permission [131]. Copyright 2023, Springer Nature. (b) Schematic of the Si PIC transmitter for polarization‐encoded QKD. Reproduced with permission [149]. Copyright 2016, Optica Publishing Group. (c) Schematic of the single‐photon frequency conversion chip based on the LN platform. Reproduced with permission [166]. Copyright 2023, Springer Nature. (d) Schematic of heterogeneous integrated SNSPD on photonic chips. Reproduced with permission [177]. Copyright 2021, Springer Nature.

The low key rate of quantum network is one of the main limits to its practical application, in which photon sources with higher brightness are always a chasing goal, because it can lead to a higher key rate. Compared to bulk crystal, the entangled photon sources (EPS) on chip can boost the brightness, benefiting from the strong mode confinement in the wavelength‐scale optical waveguide. In a silicon‐based platform, an entangled photon pair can be generated by spontaneous four‐wave mixing in a straight waveguide [119, 120], or more efficiently in microresonators [121, 122]. With different types of configuration, the entangled photons can be encoded in different degrees of freedom, including polarization [123, 124], time‐bin [125], energy‐time [126], path [127, 128], etc. Efforts have also been made to integrate the pump lasers on the chip by heterogeneous integration [129, 130], so that the EPS and the pump laser can be integrated in a single chip, as shown in Figure 8a [131]. In the future, such a bright EPS chip can be used on a space platform to improve the key rates in a free‐space quantum network.

Besides entangled photon sources, genuine single photon source (SPS), which emit one and only one photon at a time, are also crucial for ensuring the absolute security of QKD [132] and enabling functions such as quantum computing [133]. For the generation of such SPS, quantum dots (QDs) are an important choice, which leverage internal electron transitions to efficiently and stably output an indivisible photon within a specific time window [134]. It can generate SPS at a high rate while maintaining the multiphoton emission at a low level, hence it might be a suitable choice for a quantum network. With the advances in their fabrication in the past decades [135, 136, 137], QDs have recently achieved breakthroughs in practical applications. For instance, OD‐based photon sources have been used in fiber‐based QKD [138] and in relay nodes to extend the distance of quantum links [139, 140]. For multifunction integration toward full integration, many achievements have also been made in heterogeneous‐integrated quantum dot SPS chip in recent years, paving its way for an active quantum photonic chip toward application in free‐space quantum network in the future [141].

Another important type of SPS is the heralded single photon source (HSPS), which uses one photon of a photon pair as the trigger, so that the generation of the other photon can be heralded deterministically [134]. Owing to the advantages such as excellent single‐photon properties and low vacuum pulse probability of the HSPS, it has been successfully employed in QKD based on different protocols, such as the BB84 protocol [142, 143, 144] and the MDI protocol [145, 146, 147]. Such photon sources are typically generated through the spontaneous second‐order and third‐order nonlinear processes, also known as the spontaneous parametric down‐conversion (SPDC) and spontaneous four‐wave mixing process (SFWM) nowadays. So far, most HSPS are generated by bulky optical setups [148]. In the future, with the development of integration technology, such HSPS are expected to be a competitive option for SPS in free‐space quantum networks.

For real quantum tasks, the QKD chips were the first to be applied in real quantum links. In 2016, Ma et al. presented a transmitter for polarization‐encoded QKD on a silicon‐based chip, and used it in a proof‐of‐concept demonstration of the BB84 QKD protocol over a 5 km‐long fiber link, as shown in Figure 8b [149]. As the technology has advanced, QKD encoding/decoding chips with various encoding schemes [150], different protocols [151], and higher data rates [152] have been reported. In the short term, such QKD chips might be the first to be used in free‐space quantum network. In the long term, quantum chips for other quantum information tasks such as quantum computing [153], quantum teleportation [154] might also be used in a free‐space quantum network, for application in distributed quantum computing, quantum sensing, etc [155].

Besides the silicon‐based platform, many other materials have also been used for integrated quantum devices [156, 157], including III‐V material, chalcogenide glass (ChGs) material, etc. Among these platforms, the lithium niobate (LN) platform, regarded as “silicon for photonics”, features exceptional optical properties including a broad optical transparency window, high nonlinearity and electro‐optic coefficients, etc., which is a competitive candidate for quantum photonic chips [158, 159]. In LN waveguides, record‐high SPDC photon pairs have been generated [160, 161, 162], which might bring resolution in key rate if applied in the space platform in the future. As shown in Figure 8c, it can also be used for efficient single‐photon frequency conversion [163, 164, 165, 166], which can be used for applications such as up‐conversion detectors [167] and interconnection between quantum storage and quantum communication [168, 169, 170, 171] in an airborne platform in the future. The strong electro‐optic effect in LN has also been used for high‐speed and high‐fidelity manipulation of photon states [168, 172, 173, 174, 175]. Leveraging this capability, high‐performance quantum photonic chips, including but not limited to QKD chips with unprecedented high key rates, can be expected in the future. With breakthroughs in these quantum photonic chips and their application on satellites and mobile platforms, a powerful multifunction free‐space quantum network might be established in the future.

Additionally, the integration of SPD is also crucial for reducing the weight of payloads on satellites and mobile platforms. In recent years, advances in heterogeneous integration technology have enabled the integration of SNSPDs and single‐photon avalanche diodes (SPADs) on photonic chips, as shown in Figure 8d [176, 177, 178, 179, 180, 181]. For future application on these moving platforms, there is a long way to go for the development of integration SPD, in efficiency, reliability, miniaturization of cooling cryostats, and many other areas.

So far, the quantum chips are focusing on optical integration. In the future, the electronic control circuits also need to be integrated together with the optical chip, for the development of a full functional compact, and robust quantum payload. Such a low size, weight, and power (SWaP) quantum payload can be used on flying platforms, and greatly improve performance metrics such as stability and bandwidth. It can also be adopted in ground stations, advancing the development of portable ground terminals to gain flexibility and cut costs. Such technology can lay as the equipment foundation for the practical application of free‐space quantum networks.

#### 5.2.2 Acquisition, Pointing, and Tracking System

Another key technology for all free‐space quantum networks is the APT system. It is an active closed‐loop feedback system, which can achieve and maintain high‐precision and high‐stability dynamic optical pointing between terminals, for the establishment of low‐loss, high‐fidelity, and high signal‐to‐noise ratio (SNR) free‐space quantum links. To minimize the tracking error loss for longer working distances, higher tracking accuracy is always the priority for the APT system. In applications for flying platforms such as balloons [21, 182], airplanes [101, 183], and helicopters [102] toward satellites [12, 17, 18, 97], the integration and robustness of the system are also needed. In this area, various APT systems with optical diameters ranging from tens to hundreds of millimeters and weights ranging from several kilograms to hundreds of kilograms have been developed, to establish quantum links with a maximum distance of over 1000 kilometers.

In the future, the integration and miniaturization of the APT system will also be necessary. It is of particular significance for the quantum network, because, due to the point‐to‐point nature of light, the maximum number of links a free‐space quantum node can provide is determined by the number of APTs the host can load. For a short‐distance quantum network, the optical aperture of the optical antenna can be properly reduced to achieve an optimized balance between system size and link efficiency. For a long‐distance quantum network, however, a sufficiently large optical antenna is needed because of the principle of Gaussian beam diffraction [184, 185], which is not the primary choice for miniaturization.

On the other hand, the components in the post‐processing circuit are mostly conventional commercial bulk optical, electrical, and mechanical devices, which can be replaced by new‐type integrated or even chip‐scale devices for better integration. For instance, Huang et al. from Shanghai Institute of Microsystem and Information Technology uses AlScN piezoelectric MEMS mirror rather than the conventional mechanical gimbals for coarse tracking, which drastically reduces the system size and weight, and successfully used it in drone‐based applications [186]. Besides the mechanical mirror, the optical beam steering can also be realized by phase modulation of the beam wavefront, which can be realized by integrated devices like optical phased arrays [187] and liquid crystal phase modulation arrays [188, 189]. Such integrated beam‐orientation control technology can be used to replace the bulky mechanical actuators, such as fast steering mirrors, to both lose weight and improve power efficiency.

Besides, though the reduction of optical aperture is not a brilliant idea for miniaturization in long‐distance applications, new technologies like the metasurface might be applied to reduce the size and weight of the optical antenna. Such metalens uses 2D sub‐wavelength structures to modulate the phase, intensity of the beam wavefront, hence can revolutionize the reduction of the weight of the optical antenna [190]. In the future, by adopting the above technology for the APT in a space platform, the size, weight of the APT system can be significantly reduced, which can enable more APTs to be loaded on a single host so that more quantum links can be established with the same node toward a practical multi‐node free‐space quantum network.

The link loss caused by atmospheric turbulence is another important problem in free‐space quantum network [191]. It causes random variations in the refractive index of the atmosphere, which in turn induces beam spreading and wander, intensity scintillation, and wavefront distortion on the transmitted photons. These effects not only reduce the coupling efficiency to the signal fiber, but also degrade the fidelity of quantum states [53, 192]. Currently, the primary method to mitigate the adverse effects of atmospheric turbulence is adaptive optics [53, 193, 194, 195, 196]. In 2025, Scarfe et al. used high‐speed adaptive optical systems (Bertin Alpao AO kits) in counteracting atmospheric turbulence. It improved the single‐mode fiber coupling efficiency of Gaussian beams from 36.6% to 87.1%, and restored the channel fidelity of orbital angular momentum (OAM)‐encoded signals from less than 50% (under turbulence) to over 95% [197]. So far, the current AO systems are bulky and heavy, hence can only be used in fixed ground stations. In the future, for atmosphere turbulence compensation in air‐to‐ground quantum links, efforts need to be made to develop compact and lighter AO systems.

Additionally, atmospheric absorption and scattering present significant challenges in the free‐space quantum network, especially under adverse weather conditions like fog, haze, rain, and snow [198]. It is a common problem that all three types of free‐space quantum networks face, so long as they need to connect to end‐users at ground, which severely limits the time window and hence the practicality of the free‐space quantum network. To solve these problems, a multi‐node topological architecture, combining the satellites and various types of mobile platforms like drones, balloons, airships, etc., is needed. So that, a long‐distance free‐space quantum network can be established air‐to‐air without limit from the atmosphere or Earth's curvature. For air‐to‐ground transmission, a short free‐space downlink to a ground station can be established dynamically via the closest flying node to minimize the free‐space link within the atmosphere. For this task, the captive balloons might also be a promising choice, which can provide a fiber‐based downlink. So that the influence of the atmosphere can be eliminated and reliable quantum links can be established even in harsh weather. Using such configuration, full‐time all‐location coverage can be achieved with enormous network capacity, which can satisfy the demand of massive end‐users all around the world.

#### 5.2.3 Quantum Relay and Storage

Besides the quantum payloads and APT systems, the quantum relay and quantum storage also provide necessary functions for free‐space quantum networks. Quantum relays are the intermediate nodes in quantum networks, which can extend the distance for QKD and information transmission by dividing a long quantum channel into shorter segments. Unlike classical relays, which amplify signals destructively, quantum relays preserve coherence through quantum operations such as entanglement swapping and quantum teleportation, eliminating the need for trusted nodes. On the other hand, quantum memories can store, preserve, and retrieve quantum states of photons them without destroying their quantum information. It can address the asynchronicity in quantum networks by storing and releasing photon states on demand, which can serve as interfaces between flying qubits (e.g., photons) and stationary qubits (e.g., atoms or solid‐state spins). In this area, a lot of advances have been achieved in recent years, not only in‐lab [199, 200], but also for ground‐based experiments [201]. It is worth noticing that portable quantum memory has also been developed recently by Jutisz et al. [202], which achieves high efficiency and fidelity in non‐laboratory conditions. Hopefully, with further improvements, these technologies can be used in satellites and mobile platforms, and finally toward deployment in a practical free‐space quantum network in the future.

### 5.3 Network Architecture and Protocols

With advances in the above enabling technologies, breakthroughs can be expected among the ground, satellite, and mobile platform‐based quantum networks. To combine them organically toward a multilayer multi‐node practical quantum network in the future, there are numerous relevant achievements that can be learned from the fiber‐based quantum networks. First and foremost, to satisfy the interconnection requirement among multiple uses, abundant photon resources are the prerequisite, together with a network protocol for efficient quantum resource deployment. It can be achieved by different DoFs, including energy‐time, time bin, OAM, path, etc.

**FIGURE 9:**
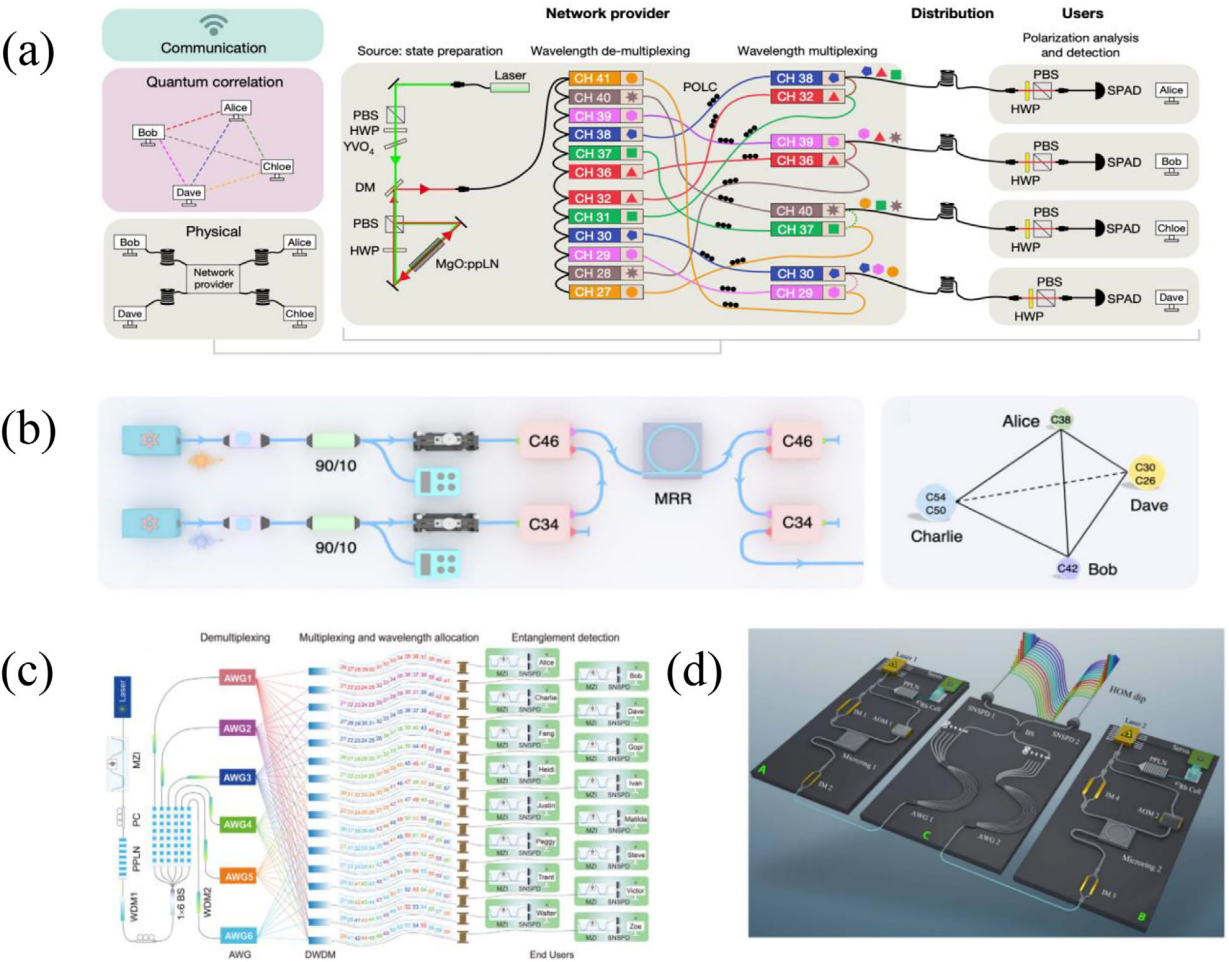
a) Architecture and experimental set‐up of the fully connected 4‐user quantum network. Reproduced with permission [208]. Copyright 2018, Springer Nature. (b) Generation of correlated photon pairs with dual pumps (left) and communication layer and wavelength allocation (right). Reproduced with permission [212]. Copyright 2025, Springer Nature. (c) Experimental setup for the sixteen‐user fully connected QKD network. Reproduced with permission [213]. Copyright 2025, Wiley‐VCH. (d) Principle of massively parallel channel HOM interference based on two standalone soliton microcombs. Reproduced with permission [218]. Copyright 2025, American Association for the Advancement of Science.

Among these DoFs, the energy‐time is the easiest choice, as it can leverage wavelength‐division multiplexing (WDM) technology, which has been well established in classical networks. For this application, generation of a broadband high‐dimensional energy‐time entangled photon source is the key resource, which can provide abundant spectral resources to satisfy the requirements of massive users. In 2015, Xie et al. generated the first bi‐photon quantum comb using a post‐filtering SPDC photon source [203]. They characterized the high‐dimensional entanglement in both time and frequency domains, and observed revival in both the HOM and Franson interference measurements. After this work, many works followed in generating the high‐dimensional energy‐time entanglement photon source [131, 204, 205, 206, 207] and exploring its application in a multi‐user quantum network. In 2018, Wengerowsky et al. demonstrated a fully connected 4‐user quantum network using a static, hierarchical‐tree‐based WDM architecture with passive filters, as shown in Figure 9a [208]. In 2020, Wen et al. demonstrated a multi‐user QKD network based on an integrated silicon nitride resonator‐generated QFC [209]. In 2021, Lingaraju et al. proposed a more efficient architecture, which used an active wavelength‐selective switch (WSS) to flexibly distribute the photon sources among different users on demand [210]. To tackle the quadratically increasing requirement O(*N* ^2^) of wavelength channel number for *N* users, Liu et al. proposed a reconfigurable entanglement distribution network. In this work, they used a multiple‐pump SFWM source together with a time‐sharing method, so that the number of required wavelength channels is successfully relaxed to O(*N*) [211]. In 2025, Yu et al. employed cascaded Mach‐Zehnder interferometers (MZIs) and a spiral waveguide to produce a QFC, enabling high‐dimensional BBM92‐QKD [76]. In the same year, Fan et al. established a fully connected quantum network based on a silicon nitride resonator‐based SFWM QFC, where the dual‐pump configuration was used so that only six wavelength channels are needed for the establishment of a four‐user graph network, as shown in Figure 9b [212].

Time multiplexing can also harness the rich temporal resource of photons, which has also been used in scalable quantum networks. In 2024, Huang et al. demonstrated a 16‐user fully interconnected quantum network using time‐bin entangled photons. The system leveraged an integrated PPLN waveguide source combined with dense wavelength‐division multiplexing (DWDM) to distribute 120 entangled photon pairs in the telecom band, enabling simultaneous pairwise entanglement across all users, as shown in Figure 9c [213]. Path is also another scalable DoF which can be used for a quantum network. In 2025, Liu et al. also demonstrated a 5‐user quantum network using a high‐dimensional path‐entangled photon source, which was generated using a metalens array and an ^85^Rb vapor cell [214].

Though plenty of efforts, as mentioned above, have been made in generating various types of high‐dimensional quantum resources to fulfill the multiuser requirements, such resources are still excessively precious. An efficient and dynamic resource management and deployment protocol is still needed for a large‐scale network. In 2017, Cao et al. proposed a Key on Demand (KoD) scheme for QKD‐secured software‐defined optical networks, which includes dynamic quantum key pools (QKP), and a novel routing, wavelength, and key assignment (RWKA) algorithm [215]. This research offers an efficient resource management and key allocation framework, for quantum‐secured large‐scale programmable optical networks. There are plenty of other QKD network protocols. Readers who are interested in this part can read the previous review for more details [216].

It is worth mentioning that, with the technology boost in optical frequency comb (OFC), OFC‐based WCS has become an important choice for multi‐node QKD network, as the fully stabilized optical frequency comb can provide frequency‐stabilized discrete frequency bins. It can be used to generate wavelength‐multiplexed identical photon sources, enabling Hong‐Ou‐Mandel (HOM) interference with high visibility. The HOM interference is a two‐photon quantum interference effect that occurs when two identical single photons enter a 50:50 beam splitter, one in each input port, which is the building block for MDI‐QKD. Recently, Yan et al. later showcased a wavelength‐multiplexed MDI‐QKD network using a dissipative Kerr soliton comb, achieving a fully connected multi‐user QKD network [217]. And Huang et al. experimentally verified HOM interference with 50 parallel comb‐teeth pairs from two independent fully stabilized soliton microcombs on chip, which can be used for the establishment of a large‐scale MDI‐QKD network in the future, as shown in Figure 9d [218]. Though an integrated frequency comb has been generated on chip in many works before [219, 220, 221], its full stabilization is still normally based on complex and bulky electrical servo devices, which limit the practical application in mobile platforms. In the future, with full integration for both optical and electrical components, a chip‐scale fully stabilized frequency comb can be generated and used for the establishment of multi‐user free‐space quantum networks.

Actually, the free‐space quantum network has also been conceived in some works [222, 223], yet no real demonstration has been achieved so far. The challenge in photon resources and network protocols can be resolved using technologies similar to the aforementioned fiber network, while the challenge in APT is the main constraint for a free‐space network. Unlike RF wireless communication, a free‐space quantum link can only be established point‐to‐point, with each link consuming a pair of APT systems. As a result, multiple APTs are needed in each node, which brings challenges not only in size and weight for the quantum payload, but also in multi‐target recognition, deployment, and dynamic routing technologies. And it becomes even more challenging for the satellites and mobile platforms. Generally, for practical demonstration of a multi‐node free‐space quantum network in the future, revolutionary breakthroughs are needed in both miniaturization and architecture of APT technologies.

## 6 Summary

In conclusion, a free‐space quantum network is indispensable, in parallel to the fiber quantum network, for a practical quantum network, with advantages in low loss and flexibility. For ground‐based quantum networks, different types of quantum experiments, including quantum key distribution, entanglement distribution, quantum teleportation, etc., have been demonstrated, with link distances of over a hundred kilometers. And a lot of new technologies, such as time‐bin, OAM, and CV encoding, and new protocols like MDI‐QKD, TF‐QKD have also been demonstrated in free space in recent years. The satellite‐based free‐space quantum network has also been achieved successfully, making breakthroughs in the distance for free‐space quantum links to over 1,000 kilometers. Experiments using mobile hosts, including balloons, helicopters, etc., have also been demonstrated, and the drone‐based mobile quantum network provides a flexible and reconfigurable choice for the establishment of quantum connections on demand. These state‐of‐the‐art free‐space quantum networks are all based on a simple architecture for quantum interconnection between two nodes. With breakthroughs in miniaturization and even monolithic integration for the quantum payloads and the APTs, multiple portable terminals can be deployed in a single host. Together with multiplexing and routing technologies, a flexible and efficient multi‐node topological free‐space quantum network can be expected.

In the future, combining all these three types of free‐space quantum network together, together with the fiber‐based quantum network, a space‐air‐ground‐sea integrated quantum network can be established from outer space to ground. In this architecture, satellite constellations act as the backbone for long‐distance toward global‐scale quantum connections, where high‐orbit satellites serve as core nodes connecting multiple low‐orbit satellites. Ground stations function as key interfaces between moving platforms and terrestrial infrastructure, receiving quantum signals from satellites and mobile platforms and connecting them to fiber‐based quantum networks. Fiber‐based networks further provide high‐capacity and stable backbone connections within and between cities. Mobile platforms, including drones, ground vehicles, and maritime systems, bridge the gap between backbone networks and end users, enabling plug‐and‐play connections to end‐users on demand without limitation. Through the coordination of these platforms, a hierarchical quantum network with full‐time, all‐location coverage can be realized. Such a network can satisfy the requirement for massive users all around the world, providing a key foundation for the development and practical applications of quantum information technology.

## Funding

This work was supported by the National Key R&D Program of China (No.2025YFF0524600), National Natural Science Foundation of China (62305156, 62293523, 62293520), the Fundamental Research Funds for the Central Universities (021014380250), and Postgraduate Education Reform Project of Jiangsu Province (2025JGZD090).

## Conflicts of Interest

The authors declare no conflict of interest.

## Data Availability

No new primary research results have been included, and no new data were generated or analyzed as part of this review.
